# Delayed post-traumatic stress and memory inflation of life-threatening events following a natural disaster: prospective study

**DOI:** 10.1192/bjo.2021.955

**Published:** 2021-07-13

**Authors:** Trond Heir, Ajmal Hussain, Pål Kristensen, Lars Weisæth

**Affiliations:** Section for Trauma, Catastrophes and Forced Migration, Norwegian Center for Violence and Traumatic Stress Studies, Norway; and Institute of Clinical Medicine, University of Oslo, Norway; Division of Mental Health Services, Akershus University Hospital, Norway; Center for Crisis Psychology, University of Bergen, Norway; Institute of Clinical Medicine, University of Oslo, Norway

**Keywords:** Post-traumatic stress disorder, aetiology, epidemiology, trauma, diagnostics

## Abstract

**Background:**

The causes of delayed post-traumatic stress disorder (PTSD) are debated, and the validity of late-onset PTSD has been questioned.

**Aims:**

We aimed to examine predictors of delayed PTSD in a community sample of survivors of a natural disaster.

**Method:**

Norwegian survivors of the 2004 Indian Ocean tsunami (*n* = 532) responded to a questionnaire at 6 and 24 months post-disaster. The questionnaire measured PTSD symptoms, recalled exposure and immediate stress responses to the disaster, recalled perceived life threat, personality dimensions, social support and other subsequent adverse life events.

**Results:**

When dichotomising PTSD symptom scores, 331 participants had low and 194 had high PTSD scores (early-onset PTSD) at 6 months. Of those with initially low scores, 43 (13.0%) had high symptom scores (delayed PTSD) at 24 months. The delayed PTSD group had a lower degree of initially assessed threat and witness experiences of death or suffering, lower immediate stress response and higher degree of memory inflation of perceived threat than the early-onset PTSD group. Among those with low PTSD scores at 6 months, onset of delayed PTSD was associated with neuroticism and memory inflation of life threat, but not with the degree of initially assessed disaster exposure or reports of subsequent adverse life events.

**Conclusions:**

Lack of association between trauma exposure and delayed onset of PTSD symptoms casts doubt on whether the traumatic event is actually the primary causative factor for delayed PTSD. Our findings suggest that delayed PTSD may be a manifestation of personality factors and memory inflation of the severity of an event.

Delayed-onset post-traumatic stress disorder (PTSD) has been a subject of discussion since it was included with the original definition of PTSD in the DSM-III. According to the DSM-5, PTSD with delayed expression occurs when the full diagnostic criteria are not met until at least 6 months after the traumatic event.^1^ Systematic reviews indicate that delayed PTSD accounts for approximately 25% of those who develop PTSD.^2,3^ Various theories have been put forward to explain the delay in PTSD, usually without strong evidence in favour of the explanation.^4^ The strongest evidence is for the possibility that delayed PTSD may be the result of additional stressors in the aftermath of the trauma.^5,6^ Sceptics have criticised the empirical basis for the diagnosis, as well as the fact that trauma is the main causal factor.^7,8^ Others have suggested that delayed PTSD might be a culturally bound expression.^9^ Thus, there is a need to clarify whether there are differences between trauma survivors who develop early-onset PTSD and delayed PTSD, and what characterises those who develop delayed PTSD compared with those who do not get PTSD.

## Memory amplification of life threat

In a previous follow-up study of Norwegian tourists who experienced the 2004 Indian Ocean tsunami, we showed that recalled severity of life threat increased over time in a significant proportion of the survivors.^10^ The findings led to an editorial in *British Journal of Psychiatry*, in which Greenberg and Wessely suggested that memory amplification of the severity of the event could be a causal mechanism for delayed PTSD.^11^ In the present study, we wanted to test this hypothesis by re-analysing our previous data. In addition to the changes in memory of life threat, we wanted to study PTSD risk factors established in the literature, such as trauma exposure, peri-traumatic responses, female gender, low education, unemployment, subsequent stressful life events, personality factors and lack of social support.^12,13^ We hypothesised that survivors with delayed PTSD had lower levels of trauma exposure than those with early-onset PTSD, and that they had greater memory inflation of the severity of the event than those who did not develop PTSD.

## Aim of the study

The aim of the study was to examine what characterises those with early-onset PTSD, delayed PTSD and no PTSD. Based on follow-up assessments at 6 and 24 months, we also wanted to examine predictors for the development of delayed PTSD among those with low symptom scores at 6 months.

## Method

### Study design

The study had a longitudinal design in which disaster survivors responded to a questionnaire administered at 6 and 24 months after the disaster. Data on background variables, disaster exposure and post-traumatic stress reactions were obtained.^14^

### Study population

Potential participants were Norwegian tourists over 18 years of age, who had visited the areas of Khao Lak, Phi Phi Islands, Krabi province or Phuket in Thailand, during the 2004 Indian Ocean tsunami. A total of 1511 people were eligible for our study, according to lists provided by the Norwegian police who registered all Norwegian citizens who had stayed in the disaster area during the tsunami. Of these, 643 (42.6%) responded at 6 months, 816 (54.0%) responded at 24 months and 532 (35.2%) responded at both waves of assessment. People who had visited the areas that were most severely affected had a much higher response rate, whereas those who had visited less severely affected locations had correspondingly lower response rate.^14^ Also, telephone calls to a random sample of non-respondents showed that they were less likely to have been exposed to the disaster than responders.^15^ The most common reasons reported for non-participation were limited interest or time, or being unaffected by the tsunami.

We excluded seven individuals from those who responded to both waves of assessments, because of missing data. The remaining 525 participants were similar in age (mean 44.0 years), but there was a slightly higher proportion of women (55%) than in the 986 individuals who were not included.^10^ Also, the 525 participants did not differ significantly in education level (57% had >12 years of education), employment (73% were employed), family constellations (69% were married or cohabitant) or pre-disaster contact with health professionals, compared with the 402 individuals who responded only at the first or second assessment. The stated lifetime prevalence for contact with a general practitioner, psychologist or psychiatrist for reasons of mental health before the tsunami was 23%. The study sample was like the age- and gender-adjusted Norwegian population in employment and marital status, but had a higher level of education and were more involved in family constellations with children.^16^

The study was approved by the Regional Committee for Medical Research Ethics and the Norwegian Data Inspectorate (project number 12858). All participants provided written informed consent through the questionnaire.

### Measures

#### Post-traumatic stress

We categorised participants into early-onset PTSD, delayed PTSD and no PTSD, according to whether the participants reported high or low symptom scores on post-traumatic stress at the 6- and 24-month assessments post-disaster. At both waves of assessment, we used the 22-item Impact of Event Scale – Revised (IES-R)^17^ to assess post-traumatic stress symptoms during the past week. Based on their experiences with the tsunami, the participants responded on a five-point Likert scale (0–4). We used the total score of IES-R as a semi-continuous measure of symptom severity, in the possible range 0–88. We replaced a missing response with a score from the same subject on another item that, on the sample level, had the highest correlation coefficient (*κ*-value) with the item that was missing. We excluded participants from analyses when they had ≥ 25% missing data (*n* = 7). High versus low PTSD symptom scores were defined as whether the participants had an IES-R score of ≥33, which is a recommended cut-off for the best diagnostic accuracy of PTSD.^18^

#### Exposure level and the immediate disaster response

The 6-month questionnaire included a wide range of disaster exposures, such as whether the respondent was caught, touched or chased by waves; sustained physical injuries; witnessed experiences of death or human suffering; or had a close relative or friend who died.^14^ Participants were also asked whether their immediate responses to the disaster were characterised by fear, helplessness or horror.^10^ Responses were measured on a five-point scale from not at all (0) to intense (3) and extreme (4). A score of 3 or 4 was considered as a positive response.^10^

#### Recalled perceived life threat

At both waves of assessment, we measured the threat intensity of the original exposure by the question: ‘How great do you think the danger was that you would die?’ Responses were given on a five-point scale: none (1), small (2), moderate (3), great (4) or overwhelming (5).^10^ We specified change in recalled intensity of life threat from 6 to 24 months as the score at 24 months minus the score at 6 months.

#### Neuroticism

Neuroticism was measured with the 44-item Big-Five Inventory,^19^ which was scored on a seven-point scale from 1 (does not fit) to 7 (fits entirely). In a *post hoc* evaluation of whether variability in neuroticism reflected trauma exposure or antecedent risk for PTSD, we examined the correlations between disaster exposure and the scores of neuroticism, which revealed low and non-significant correlations (‘caught, touched or chased by waves’: Spearman's *r* = 0.05, *P* = 0.25; ‘witnessing dead bodies’: Spearman's *r* = 0.02, *P* = 0.70; and ‘loss of family member or close friend’: Spearman's *r* = 0.02, *P* = 0.70), indicating that assessments of neuroticism were negligibly affected by trauma exposure.

#### General self-efficacy and social support

We used the ten-item General Self-Efficacy Scale^20^ to measure self-beliefs in coping with demands, tasks and challenges of life in general, and the six-item Crisis Support Scale^21^ to measure social support after the disaster. We analysed negative social responses, support satisfaction and positive social support separately.^22^ Self-efficacy items were scored on a four-point scale (1, completely wrong; 2, quite wrong; 3, quite right; 4, completely right), whereas social support items were scored on a seven-point scale, from 1 (never) to 7 (always).

#### Other life events

We used the 12-item Life Events Inventory^23^ to assess negative life events experienced in the past 12 months. We also included an additional but separately analysed item: whether the participants had experienced a severe accident, armed robbery, physical violence, rape, war or disaster in the past 12 months.^10^

### Statistical analyses

We classified participants into three symptom groups according to symptom assessments at 6 and 24 months post-disaster: early-onset PTSD, delayed PTSD and no PTSD. Participants were classified as having early-onset PTSD if they had a high symptom score (IES-R≥33) at the 6-month follow-up; delayed PTSD was defined as having a low symptom score (IES-R<33) at the 6-month follow-up and a high symptom score at the 24-month follow-up; and no PTSD was defined as having low symptom scores at both assessments.

We performed *χ*^2^-tests with subsidiary Bonferroni-corrected *post hoc* tests to compare the three allocated symptom groups with regards to categorical demographic variables, different types of exposure and peri-traumatic responses. We used one-way ANOVAs with subsidiary Bonferroni-corrected *post hoc t*-tests to compare the allocated groups regarding age, life-threat intensity, personality dimensions, self-efficacy and social support. The distribution of these variables was found to be sufficiently close to the normal distribution for such analyses.^24^

Variables that showed a significant difference between the delayed PTSD group and the no PTSD group were considered as potential predictors of delayed PTSD. This ended up being three variables, all of which were independently subjected to logistic regression in the subpopulation of individuals with initially low PTSD symptom scores. We used the development of delayed PTSD as a dependent variable. We used multiple logistic regression analysis to study adjusted effects of these variables, along with gender and age. We used two-tailed tests and set the significant level at *P* = 0.05. Statistical analyses were carried out with the software package SPSS, version 18.0 for Windows.

## Results

**Fig. 1:**
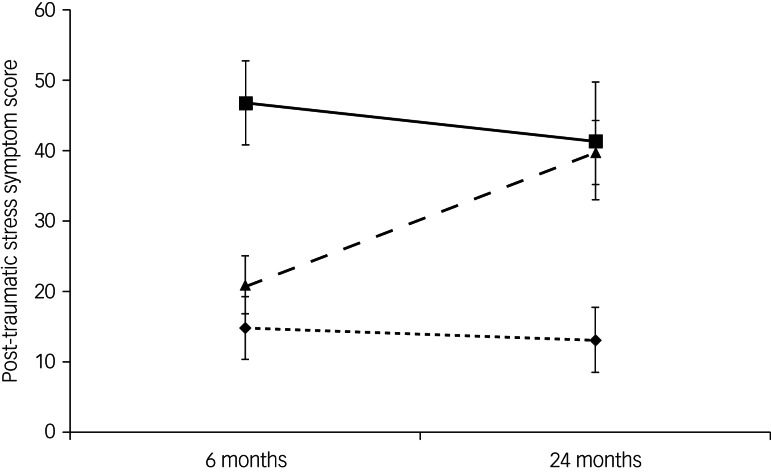
Post-traumatic stress symptom score (mean total Impact of Event – Scale Revised score with s.d.) in tsunami survivors classified as having early-onset PTSD (squares with solid line; high score at 6 months, *n* = 194), delayed PTSD (trangles with dashed line; low score at 6 months and high score at 24 months, *n* = 43) and no PTSD (diamonds with dotted line; low score at both time points, *n* = 288). Cut-off for high versus low scores was ≥ 33. PTSD, post-traumatic stress disorder.

Of the 525 participants who completed both waves of assessment, 331 (63%) had low and 194 (37%) had high scores (early-onset PTSD) at the 6-month follow-up. Of the 331 participants with low PTSD symptom scores at 6 months, 43 had high scores at the 24-month follow-up (delayed PTSD). Delayed PTSD accounted for 18.1% of all participants who had high symptom scores at either follow-up. Figure 1 shows the trajectory of PTSD symptom scores in three groups according to whether high symptom scores were early-onset PTSD, delayed PTSD or no PTSD.

Significant differences between the three allocated groups were found for gender and employment (Table 1). The early-onset PTSD group had a higher proportion of women compared with the no PTSD group. Both the early-onset and delayed PTSD groups had a higher proportion of unemployed participants than the no PTSD group.

**Table 1:** Demographic data of 525 disaster-exposed Norwegian tourists, according to onset of PTSD after the 2004 Indian Ocean tsunami

	Early-onset PTSD (*n* = 194)	Delayed PTSD (*n* = 43)	No PTSD (*n* = 288)	Test value *F*(2, 522) or *χ*^2^
Age, years, mean (s.d.)	44.0 (13.4)	46.1 (14.7)	43.6 (12.3)	0.68
Gender, female	130 (67.0)	24 (55.8)	138 (47.9)	17.12***
Married or cohabiting	130 (69.9)	25 (64.1)	195 (70.1)	0.60
Education >12 years	103 (53.1)	21 (48.8)	177 (61.7)	4.91
Employed	121 (62.4)	26 (60.5)	234 (81.3)	24.20***

Demographic data refers to status at 6-month follow-up. Unless otherwise stated, variables are given as *n* (%). The early-onset PTSD group had a higher proportion of women compared with the no PTSD group. Both early-onset and delayed PTSD groups had a lower proportion of employed respondents than the no PTSD group. PTSD, post-traumatic stress disorder.

*** *P* < 0.001.

### Disaster exposure and peri-traumatic responses

The early-onset PTSD group had been more severely exposed to every type of disaster stressor than either of the two groups that did not have high symptom scores at 6 months (Table 2). The early-onset PTSD group had also been more prone to fear, helplessness and horror during the event. There were no significant differences between the delayed PTSD group and the no PTSD group either in disaster exposure or peri-traumatic responses.

**Table 2:** Disaster exposure and peri-traumatic responses recalled at 6 months post-disaster in disaster survivors, grouped according to onset of PTSD

	Early-onset PTSD (*n* = 194)	Delayed PTSD (*n* = 43)	No PTSD (*n* = 288)	Test value	Effect size
	*n* (%)	*n* (%)	*n* (%)	*χ*^2^	Cramér's *V*
Disaster exposure					
Caught/touched/chased by waves	108 (55.7)	15 (34.9)	92 (32.6)	25.98***	0.22
Physical injury inflicted	40 (21.6)	3 (7.5)	27 (9.5)	15.19**	0.17
Witness to dead bodies	93 (52.0)	16 (38.1)	91 (32.2)	18.00***	0.19
Witness to survivors with serious physical injuries	139 (76.0)	24 (60.0)	156 (55.7)	19.77***	0.20
Witness to abandoned children	88 (48.6)	9 (22.5)	65 (23.5)	33.55***	0.26
Loss of family member or close friend	25 (13.1)	1 (2.3)	13 (4.6)	13.78**	0.16
Peri-traumatic responses					
Fear	135 (75.0)	18 (45.0)	128 (46.0)	39.57***	0.28
Helplessness	146 (80.7)	21 (53.8)	139 (50.0)	44.54***	0.30
Horror	18 (10.7)	1 (2.7)	6 (2.3)	15.06**	0.18

The early-onset PTSD group reported higher exposure to every disaster stressor and peri-traumatic response than either of the other groups. There were no significant differences between the delayed PTSD group and the no PTSD group. PTSD, post-traumatic stress disorder.

**P < 0.01, ***P < 0.001.

### Recalled intensity of perceived life threat

The early-onset PTSD cases reported a higher degree of life threat at 6 months than either of the two other groups, which did not differ significantly from each other (Table 3). From 6 to 24 months there was an overall memory inflation of perceived life threat, which was most extensive in the delayed PTSD group. When reported at 24 months, there was no significant difference between the early- and delayed-onset PTSD cases. Both of these groups recalled a higher degree of life threat than the no PTSD group at the 24-month assessment.

**Table 3:** Recalled intensity of life threat at 6 and 24 months post-disaster in groups of tsunami survivors according to onset of PTSD

	Early-onset PTSD (*n* = 194)	Delayed PTSD (*n* = 43)	No PTSD (*n* = 288)	Test value	Effect size
	Mean (s.d.)	Mean (s.d.)	Mean (s.d.)	*F*(2, 522)	*ŋ^2^*
Recalled intensity of life threat at 6 months	2.20 (1.39)	1.36 (1.43)	1.25 (1.27)	29.30***	0.10
Recalled intensity of life threat at 24 months	2.62 (1.12)	2.55 (1.26)	1.84 (1.18)	22.75***	0.10
Change in recalled intensity of life threat from 6 to 24 months	0.31 (0.80)	1.00 (0.95)	0.42 (0.85)	8.66***	0.04

Threat intensity was based on the question ‘How great do you think the danger was that you would die’, measured on a scale of 0–4. PTSD, post-traumatic stress disorder. The early-onset PTSD group recalled a higher degree of life threat at 6 months than either of the other groups. At 24 months, both the early-onset and delayed PTSD groups recalled a higher degree of life threat than the no PTSD group. The change in recall intensity of life threat from 6 to 24 months was higher in the delayed PTSD group than in the other groups.

*** *P* < 0.001.

### Personality factors and social support

Both the early-onset and delayed PTSD groups had higher levels of neuroticism than the no PTSD group (Supplementary Table 1 available at https://doi.org/10.1192/bjo.2021.955). Assessments of perceived social support were not significantly different in the delayed PTSD group compared with the no PTSD group (Supplementary Table 1). However, the early-onset PTSD group reported lower levels of positive social support, support satisfaction and self-efficacy than the no PTSD group, and higher levels of negative social response. The early-onset PTSD group also had lower general self-efficacy, whereas there was no difference in self-efficacy between the delayed PTSD group and the no PTSD group.

### Additional stressors

At the 24-month assessment, the three symptom groups differed significantly in negative life events experienced during the previous 12 months (*F* = 6.18, *P* < 0.01). However, the number of such events in the delayed PTSD group (mean 1.2, s.d. 1.4) did not differ significantly from what was reported by the two other groups, whereas the early-onset PTSD group reported a higher number of events (mean 1.5, s.d. 1.5) than the no PTSD-group (mean 1.0, s.d. 1.3). There were no differences between the three groups in terms of more severe events, such as severe accident, armed robbery, physical violence, rape, disaster or war incidents (*χ*^2^ = 1.99).

### Predictors of delayed PTSD

For participants who had low symptom scores at 6 months, delayed PTSD was associated with unemployment, neuroticism and memory inflation of perceived life threat in unadjusted analyses (Table 4). When adjusted for other risk factors, delayed PTSD was associated with neuroticism and memory inflation of perceived life threat.

**Table 4:** Associations between delayed post-traumatic stress disorder (dependent variable) and employment, neuroticism and memory inflation of perceived life threat, along with age and gender (independent variables), among disaster survivors with initially low symptom scores (*n* = 331)

	Unadjusted results			Adjusted results		
	Odds ratio	95% CI	*P*-value	Odds ratio	95% CI	*P*-value
Age (increase of 10 years)	1.17	0.91–1.50	0.231	1.28	0.93–1.75	0.130
Gender (women versus men)	1.37	0.72–2.62	0.335	1.62	0.68–3.85	0.273
Employment (no versus yes)	2.83	1.44–5.59	0.003	2.18	0.88–5.41	0.092
Neuroticism (increase of one point)	1.60	1.20–2.13	0.001	1.74	1.21–2.51	0.003
Change in recalled intensity of life threat (increase of one point)	2.04	1.33–3.11	0.001	2.19	1.37–3.50	0.001

## Discussion

Our prospective study of disaster survivors showed that delayed PTSD occurred in a community sample that was exposed to a natural disaster. Compared with individuals with early-onset PTSD, those with delayed PTSD had been less exposed to disaster stressors, were less prone to peri-traumatic reactions and reported being exposed to a lower degree of life threat when assessed at 6 months post-disaster. In terms of the severity of disaster experiences, people with delayed PTSD did not differ from those who did not develop PTSD at all. However, from 6 to 24 months there was an overall memory inflation of recalled life threat, which was most extensive in the delayed PTSD group. When assessed at 24 months, there was no difference in recalled life threat between the early-onset and delayed PTSD cases. In a multiple regression analysis, which included all participants that had low symptom levels at 6 months, delayed PTSD was associated with neuroticism and memory inflation of perceived life threat.

### Trauma exposure

Consistent with the conclusions of two reviews,^2,3^ delayed PTSD accounted for a considerable part of PTSD cases. However, unlike previous studies,^5,6^ we found no evidence for the significance of additional stressors in the aftermath of the disaster. Overall, the lack of association between level of disaster exposure and development of delayed PTSD raises questions about the dose-response model of PTSD and the validity of the delayed PTSD construct.^7,8^ The limited exposure to disaster stressors was consistent across several types of trauma exposure. Among these, physical injuries and loss of close relatives are relatively factual and objectified information that should be less prone to recall bias.^14^ Our findings contradict results from a study of PTSD in war veterans,^25^ in which delayed PTSD and early-onset PTSD groups reported equal levels of war-zone trauma exposure and peri-traumatic reactions. However, in that study, symptom onset dates, trauma exposure and peri-traumatic stress responses were reported retrospectively after the development of delayed PTSD, in some cases many years after the war experiences. The timing at which we assessed trauma exposure and peri-traumatic stress (i.e. before the expression of delayed symptoms) may be of importance in the interpretation of between-study differences, as trauma memories may inflate with time.^26^

### Recalled life threat

As postulated by Greenberg and Wessely,^11^ delayed PTSD was associated with memory amplification of life threat. When a person's memory changes over time, it is not obvious which memory best represents the objective event.. However, the agreement between the delayed PTSD group's low level of recalled life threat and their reports on a broad spectrum of trauma-related variables gives strong support to the validity of their first report. Later, memory inflation may have occurred through a variety of processes, such as reappraisal or sensitisation.^27,28^ The interaction between the event characteristics and the memory processes may have been influenced by personality factors, secondary gains or factors related to the current needs of an individual.^29,30^ Threat could have been exaggerated depending on how significant the event was in terms of a person's identity, and whether it was assigned a central role in his or her life history.^31^ For example, expectations of reparative efforts by authorities might render survivors vulnerable to deception and frustration at later stages.^3^ Also, memories could have been reconstructed through media exposure or identification with more severely exposed survivors.

Many studies have shown that subjective measures of perceived threat have predicted PTSD symptoms more precisely than have objective measures of danger.^32–34^ Our findings suggest that not only is appraisal of threat a key determinant of PTSD symptoms, but changes in appraisal may also affect the course of symptoms long after the event. This agrees with the mnemonic model, which argues that it is the current memory of the event that determines whether symptoms occur.^35^ According to this view, there is no authentic memory of the original encoding that can be restored, but rather a selective, current memory that can be changed.

Theoretical explanations for the association between delayed PTSD and memory inflation of life threat should also include the possibility of a reverse causality, i.e. that exacerbation of PTSD symptoms may drive memory enhancement.^28,36^ The processes of remembering may be influenced by symptoms that move negative attention or emotions toward the event. In that case, the study provides few clues as to what drives the late onset of PTSD symptoms.

### Neuroticism

Neuroticism remained a strong predictor for the development of delayed PTSD after adjustment for other risk factors. Increased access to emotionally negative information may increase the vulnerability to delayed PTSD, similar to what has been proposed as a possible explanation for depression.^37^ In the same way as the early-onset PTSD group, the delayed PTSD group was less likely to be employed than the no PTSD group. This is in accordance with findings among war veterans.^25^ The association may be because of personality or cognitive resources of employed individuals versus those who are not employed, or employment may protect against mental distress by providing some sort of resilience. When adjusted for neuroticism, unemployment was not a significant predictor of delayed PTSD, indicating the importance of the personality.

### Study benefits and limitations

Methodological benefits include the invitation of an entire disaster population of Norwegian tourists who were exposed to the same disaster event. Potential traumatisation was less likely to be related to secondary disaster stressors such as loss of home or livelihood. The participants had above-average education and socioeconomic status, and fewer reimbursement motives than war veterans.^38^ Regardless of diagnoses, survivors were offered free medical and psychosocial care by the Norwegian state.^39^

The study has several limitations. First, there were limited response rates. Lack of participation was primarily related to the experience of personal irrelevance, such as no disaster exposure.^15^ Nevertheless, it must be considered that there may be a response bias of unknown magnitude and direction. Second, we only used self-reported measures, and data on disaster exposure and the immediate stress responses were ascertained retrospectively at 6 months post-disaster, which allows for response bias. Assessments of life-threat intensity were based on a rough measure limited to a single item; the measure of change therefore had poor sensitivity and the effect sizes were small. Third, we did not have access to PTSD diagnoses based on clinical interviews. Still, we believe the study provides valid conclusions about a significant increase in the PTSD symptom level that happened at least 6 months after exposure to the disaster. Fourth, as our measurement of neuroticism was done post-disaster, it may be questioned whether elevated scores were affected by trauma exposure or represented an antecedent risk for PTSD. However, low and non-significant correlations between disaster exposure and neuroticism indicate that elevated scores of neuroticism represent primarily an antecedent risk for delayed PTSD rather than being a result of trauma exposure. This agrees with the theory that neuroticism can be identified early in life, and shows stability over time.^40^

### Implications

Verification of a traumatic event is critical when determining a PTSD diagnosis. Also, it is a basic principle in theories of PTSD that the degree of exposure is associated with the likelihood of developing the disorder.^13,41,42^ The lack of association between exposure level and development of delayed PTSD symptoms in our study may raise questions about the appropriateness of the A1 criterion,^43^ and whether the basis for diagnosis should be the traumatic load of a real event as opposed to memories, which may have acquired traumatic characteristics. Subsequent treatment planning, healthcare rights and disability payments, among other issues, will be affected by such choices. Concerned about the validity of the delayed PTSD construct, Spitzer et al^8^ have proposed tightening the PTSD criteria, limited to cases in which ‘the symptoms develop within a week of the event’, or ‘if delayed onset, the onset of symptoms is associated with an event that is thematically related to the trauma’. Such restrictions would undoubtedly reduce the number of patients with delayed PTSD.

Memory processes that amplify the severity of an event raise questions about the extent to which cultural factors in society, healthcare, and compensation systems influence how people relate to various adverse events. Symptoms of PTSD are higher among individuals who assign a trauma as a turning point in their life story or a central component of personal identity.^44,45^ Attributing the trauma to a more important role in the individual's life may also influence the trajectory of post-traumatic stress reactions.^46^ More longitudinal studies should be conducted to explore the meaning of memory disturbance in such a process.

Health professionals should be aware of dilemmas by promoting the severity of distressing life events. In cognitive therapy, the moderation of negative appraisals of a traumatic event is a recommended approach in PTSD treatment.^47^ The preoccupation with trauma may have an opposite effect. Thus, too much attention to serious life events and their harmful effects on mental health may contribute to higher levels of mental illness in a population.^48^

In conclusion, our findings suggest that delayed onset of clinical levels of PTSD symptoms may not be related to the degree of trauma exposure itself. It seems that memory processes that affect the current recall of the exposure may be of more importance. Additional prospective research should explore how memories may acquire more traumatic characteristics, and examine the influence of social norms, culture and attitudes in this regard.

## Data Availability

Data are from the ‘Tsunami research program’, conducted by The Norwegian Centre for Violence and Traumatic Stress Studies. According to the approval from the Norwegian Regional Ethics Committee, the data is to be stored properly and in line with Norwegian privacy protection law. Public availability would compromise privacy of the respondents; however, anonymised data is freely available to interested researchers upon request to corresponding author and project leader, T.H., pending ethical approval from our ethics committee.

## Supplementary material

For supplementary material accompanying this paper visit http://dx.doi.org/10.1192/bjo.2021.955. click here to view supplementary material
