# Supplementary material for: Delayed post-traumatic stress and memory inflation of life-threatening events following a natural disaster: prospective study
Source: BJPsych Open. 2021 Jul 13;7(4):e132. doi: 10.1192/bjo.2021.955 (PMC8281038; doi:10.1192/bjo.2021.955)
Supplement: Supplementary file 1 [file S2056472421009558sup001.doc]

*Supplemental table 1. Social support and self-efficacy at 6 months post disaster in groups of disaster survivors according to onset of PTSD. The first three dimensions are from the* *Crisis Support Scale; scale 1-7. The fourth and last is from the* *General Self-Efficacy Scale; scale 1-4.*

|  | Early-onset PTSD (n=194) | Delayed PTSD (n=43) | No PTSD (n=288) | Test value | Effect size |
| --- | --- | --- | --- | --- | --- |
|  | *Mean (SD)* | *Mean (SD)* | *Mean (SD)* | *F*(2, 520) | *ŋ^2^* |
| Neuroticism^1^ | 3.89 (1.16) | 3.62 (1.19) | 3.03 (1.06) | 35.14 *** | 0.12 |
| Positive social support^2^ | 4.82 (1.38) | 5.26 (1.62) | 5.39 (1.37) | 9.83 *** | 0.04 |
| Negative social response^2^ | 2.87 (1.96) | 2.63 (2.21) | 2.21 (1.74) | 7.13 ** | 0.03 |
| Support satisfaction^2^ | 4.89 (1.68) | 5.37 (1.68) | 5.90 (1.33) | 24.20 *** | 0.09 |
| Self-Efficacy^3^ | 2.96 (0.45) | 3.18 (0.37) | 3.23 (0.43) | 23.35 *** | 0.08 |

** p<0.01. ***p<0.001. Both early- and delayed-onset PTSD groups had higher levels of neuroticism than the no-PTSD group. The early-onset group reported lower levels of positive social support, support satisfaction and self-efficacy than the no-PTSD group, and they reported higher levels of negative social response. The delayed PTSD group did not differ significantly from the no-PTSD group in any of these measures.

^1^ Big-Five Inventory (scale 1-7); ^2^ Crisis Support Scale (scale 1-7); ^3^ General Self-Efficacy Scale (scale 1-4).
